# Urine antibiogram and antimicrobial susceptibility pattern in a provincial hospital of Nepal: a cross-sectional study

**DOI:** 10.1186/s12879-026-13781-x

**Published:** 2026-06-08

**Authors:** Prashant Kumar Jha, Sachita Barma, Prabhat Kumar Jha, Biswash Sapkota, Sailendra Chaudhary, Binita Ghimire, Archana Singh, Bishnu Khakurel, Prashant Bidari, Prabin Pathak, Bipindra Pandey

**Affiliations:** 1 Department of Laboratory Medicine, Hetauda Hospital, Madan Bhandari Academy of Health Sciences, Hetauda, Bagmati Province Nepal; 2 Department of Pharmacy, Madan Bhandari Academy of Health Science, Hetauda, Bagmati Province Nepal; 3 Department of Molecular Lab and Microbiology, Hetauda Hospital, Madan Bhandari Academy of Health Sciences, Hetauda, Bagmati Province Nepal; 4 Department of Anaesthesia and Critical Care, Hetauda Hospital, Madan Bhandari Academy of Health Sciences, Hetauda, Bagmati Province Nepal; 5 Department of Pathology, Institute of Medicine, Tribhuvan University, Kathmandu, Nepal

**Keywords:** Antimicrobial susceptibility testing, Bacterial isolates, Multidrug resistance, National Action Plan, Nepal

## Abstract

**Introduction:**

The National Action Plan on antimicrobial resistance (AMR) strategic priority of Nepal focuses on strengthening the surveillance system for the containment of AMR within the country, as such, this study aims to report the prevalence of bacterial isolate and antimicrobial susceptibility pattern.

**Method:**

A hospital-based retrospective cross-sectional study was conducted using routine urine culture and antimicrobial susceptibility records from Hetauda Hospital, Nepal, from May 28, 2023, to September 8, 2023. Clinically significant uropathogens were identified using standard microbiological procedures, and antimicrobial susceptibility testing was performed using the modified Kirby–Bauer disk diffusion method. Susceptibility results were interpreted according to the CLSI M100 criteria used by the hospital laboratory during the study period. Data were analysed descriptively using frequencies, percentages, and relevant denominators.

**Result:**

Out of 3893 patient samples, 1162 (29.84%) bacterial isolates were detected. Gram-negative bacteria accounted for 67.81% of the isolates, with *Escherichia coli* (47.16%) being the most common, followed by *Klebsiella sps.* (14.54%). AST has revealed varying patterns across different antibiotics and bacterial species. *Escherichia coli* (*E. coli*) emerged as the predominant pathogen showing variable susceptibility across antibiotic classes, with highest sensitivity to amikacin (83.90%) and notable resistance to fluoroquinolones and cephalosporins, highlighting significant challenges in empirical therapy. Notably, 20.40% of the isolates were classified as MDR, with *Proteus vulgaris* showing the highest MDR rate (46.15%).

**Conclusion:**

This study highlights the prevalence of urinary bacterial isolates (*E. coli)* and multidrug-resistant strains (*Proteus vulgaris*) at Hetauda Hospital, with considerable resistance to cephalosporins and fluoroquinolones. These findings support AST-guided prescribing, local antimicrobial stewardship, and development of a hospital antibiogram. The results are contextually relevant to Nepal’s AMR policy agenda, but they should be interpreted as hospital-level data rather than as provincial or national surveillance estimates.

**Supplementary Information:**

The online version contains supplementary material available at 10.1186/s12879-026-13781-x.

## Key messages

• **What is already known on this topic** – Antimicrobial resistance (AMR) is a growing public health concern in context of developing country like Nepal, and the National Action Plan (NAP) prioritizes strengthening surveillance systems to monitor bacterial isolates and resistance patterns.

• **What this study adds** – This study provides recent hospital-based urine culture data on bacterial isolates, antimicrobial susceptibility patterns, and multidrug resistance at a provincial hospital in Nepal.

• **How this study might affect research**,** practice or policy** – The findings can inform local empirical prescribing, hospital antibiogram development, and facility-level antimicrobial stewardship in reference to the National Antimicrobial Treatment Guideline 2023.

**Supplementary Information:**

The online version contains supplementary material available at 10.1186/s12879-026-13781-x.

## Background

The global burden analysis of bacterial antimicrobial resistance (AMR) from 1990 to 2021 estimated 4.71 million deaths, including 1.27 million directly attributed to bacterial AMR, with methicillin-resistant *Staphylococcus aureus* (MRSA) ranking among the top ten leading pathogens rather than being the sole major culprit [1]. By 2050, it is projected that deaths will increase to approximately 1.91 million globally, with over 8 million deaths related to AMR [1]. The burden is particularly higher in low-resource settings and low-middle-income countries (LMICs), with an estimated 5.0 million associated deaths [2]. 

Several factors contributing to the escalating AMR issues in LMICs include inadequate surveillance systems, the injudicious use of antibiotics in crops, poultry, and agricultural products, poor regulation of antimicrobial sales and distribution, instances of self-medication, sub-optimal dosing, poor sanitation and hygiene, unplanned infection control, inadequate healthcare facilities and infrastructure, and a lack of coordinated guidelines [3–5]. 

Nepal, a low- and middle-income country (LMIC), has also developed its own National Action Plan (NAP) for 2024–2028 to address antimicrobial resistance (AMR) within the nation. This plan incorporates a three-tier system-federal, provincial, and local levels to tackle AMR issues and advance the One Health Approach (OHA) [6]. To achieve the objectives outlined in the NAP, five strategic priorities have been established: P1: enhancing awareness and understanding of AMR through various communication channels, P2: strengthening AMR-related information through effective surveillance, P3: implementing effective infection prevention and control to reduce AMR, P4: optimizing the use of antimicrobials in human, animal, and food sectors, and P5: promoting resources and innovation [5, 6]. Each of these strategic priorities has its own distinct set of objectives, interventions, and activities aimed at mitigating AMR.

The NAP strategic priority 2 focus area (5.2.1 Surveillance of AMR) aims to enhance the national AMR surveillance system across humans, food, and animals. Consequently, the strategic intervention (5.2.1.2) emphasizes the development or establishment of National AMR surveillance standards within One Health sectors. A key activity involves creating standardized protocols and methodologies for sample testing and Antimicrobial Susceptibility Testing (AST) across nearly all sectors [6]. AST entails the in vitro analysis of bacterial activity against antimicrobial agents, aiding in the identification of sensitive and resistant patterns of these agents and guiding optimal definitive therapy for improved patient outcomes [7, 8]. 

While Nepal’s NAP on AMR emphasizes surveillance, most available data is confined to a few tertiary hospitals at the provincial level. There is a significant lack of provincial-level AMR data and routine antibiograms, leaving clinicians without the evidence needed to guide empirical therapy. This study was designed as a hospital-based cross-sectional analysis of urine culture isolates and antimicrobial susceptibility patterns at Hetauda Hospital. The intended relevance of the study is twofold: first, to provide local AST data that may support empirical prescribing and antimicrobial stewardship at the facility level; and second, to contribute contextually to NAP strategic priority 2, which emphasizes standardized AMR information generation. Although the study is contextually relevant to Nepal’s National Action Plan on AMR and the National Antimicrobial Treatment Guideline 2023, it was not designed to evaluate national surveillance performance or provincial representativeness. The specific objective of this study was to describe the prevalence and distribution of clinically significant bacterial uropathogens isolated from urine specimens at Hetauda Hospital and to determine their antimicrobial susceptibility and multidrug-resistant patterns. The reference to Nepal’s National Action Plan on AMR is intended to provide policy context for local antibiogram generation; this study was not designed to measure the performance of the national surveillance system or to generate provincial or national AMR estimates.

## Materials and methods

### Study design, settings, participants

A hospital-based cross-sectional study was conducted using retrospective laboratory records from May 28, 2023, to September 8, 2023, at Hetauda Hospital, Madan Bhandari Academy of Health Sciences, Bagmati Province, Nepal. The data consisted of routine urine culture and antimicrobial susceptibility records generated as part of regular clinical diagnostic services by the microbiology laboratory of Hetauda Hospital. The authors did not prospectively generate these data; rather, the eligible laboratory records from study periods were extracted, cleaned, and analysed retrospectively for this study. The study population comprised patients of any age or sex attending outpatient or inpatient services for whom the treating clinician requested urine culture and antimicrobial susceptibility testing. Only urine specimens processed at the hospital microbiology laboratory were included, whereas specimens referred from other hospitals were excluded. Urine specimens were selected because urine culture is one of the most frequently requested bacteriological investigations in the hospital microbiology laboratory and urinary tract infections commonly require empirical antimicrobial therapy. Restricting the study to urine specimens also reduced heterogeneity related to specimen type, collection method, culture interpretation, and antibiotic panel selection. Therefore, urine isolates were considered appropriate for developing a facility-level urine antibiogram to support local antimicrobial stewardship in context of our study setting. To avoid duplicate counting, repeat urine cultures from the same patient during the same infectious episode were screened using the hospital registration number and laboratory accession number. When more than one urine culture from the same patient yielded the same organism within [14/30] days, only the first isolate was included. Repeat cultures yielding a different organism were reviewed separately and included only when they represented a distinct microbiological episode. The study is reported in accordance with the Strengthening the Reporting Of Observational Studies in Epidemiology (STROBE) checklist for cross-sectional studies [9]. 

### Study process

#### Sample collection

Urine samples were collected by using midstream clean-catch technique from patients who visited Hetauda Hospital. Patients were instructed to wash their hands before collection. Female patients were instructed to separate the labia and clean the periurethral area from front to back using clean water or a sterile swab according to the hospital collection protocol. Male patients were instructed to retract the foreskin, where applicable, and clean the glans and urethral meatus before collection. Patients were advised not to touch the inner surface of the sterile container. The initial stream of urine was discarded, and approximately 10–20 mL of midstream urine was collected in a sterile, wide-mouthed, leak-proof container to minimize contamination. After collection, each urine container was immediately labeled, tightly capped, and transported to the microbiology laboratory in a leak-proof secondary container. Specimens were processed as soon as possible, preferably within 2 h of collection. When immediate processing was not possible, samples were stored at 2–8 °C and processed within 24 h according to the hospital laboratory protocol. Leaking, unlabeled, insufficient-volume, visibly contaminated, or excessively delayed specimens were excluded from microbiological processing. Urine specimens for microbiological testing were collected and submitted to the microbiology laboratory based on clinical judgment and necessity as determined by the attending physicians, rather than through prospective monitoring of patients until testing was required. Where clinically feasible, urine specimens were requested before initiation of empirical antibiotics; however, because this was a retrospective laboratory-record study, reliable patient-level data on prior antibiotic exposure or whether antibiotics were administered before or after urine culture were not consistently available. AST reports were issued to the treating clinicians as part of routine hospital laboratory reporting. However, this retrospective laboratory-record study did not consistently capture patient-level treatment decisions, antibiotic modification after AST reporting, duration of therapy, or clinical outcomes. Therefore, correlation between antibiogram results and individual treatment success could not be assessed. The clinical significance of uropathogens was determined using semi-quantitative urine culture criteria described below, including colony count, predominance of a single organism, and exclusion of mixed growth or probable contamination [10–12].

#### Isolation and identification of bacteria

Patients from both outpatient and inpatient settings were included, and samples were collected at the time of clinical presentation as requested by the treating physician. The patients were monitored to ascertain the etiology of the infection, necessitating the collection and transfer of urine samples to the Microbiology Laboratory of Hetauda Hospital. Subsequently, upon identifying the cause of infection, the laboratory is responsible for determining the antibiotic resistance pattern, thereby enabling the physician to prescribe the most suitable and effective antibiotic for treatment [10]. 

Morning midstream urine samples were aseptically collected using sterile containers and promptly transported to the microbiology laboratory with appropriate transport media. Bacterial identification was conducted using colony characteristics, gram staining, and various biochemical tests, following established methodologies [11]. In brief, samples were cultured on selective media, including MacConkey agar, eosin methylene blue (EMB) medium, however blood agar is non-selective medium used for the cultivation of fastidious organisms. Subsequently, bacterial identification of the isolates was performed using standard microbiological techniques [11]. Semi-quantitative urine culture was performed using a calibrated loop of [0.001 mL/0.01 mL; insert actual loop volume]. After incubation, colonies were counted, and colony-forming units per millilitre were calculated by multiplying the colony count by the loop calibration factor. A single or predominant bacterial isolate meeting the laboratory colony count threshold for clean-catch urine was considered clinically significant. Growth of two or more organisms without a predominant pathogen was considered mixed growth or probable contamination and was excluded from AST analysis [11, 29].

The microbiology laboratory of the hospital is staffed by qualified microbiologists and laboratory technologists and operates under hospital-based quality standards, though it does not hold formal external accreditation. For this study, only urine specimens were processed. Bacterial identification and antimicrobial susceptibility testing were performed through a combination of conventional biochemical methods and an automated identification system (BD Phoenix™) [11]. Further bacterial isolates were identified to the species level, with *Staphylococcus aureus* differentiated from *Coagulase-negative staphylococci* through slide and tube coagulase tests, supported by automated system confirmation [11]. 

#### Antimicrobial susceptibility testing (AST)

Antimicrobial susceptibility testing was performed using the modified Kirby–Bauer disk diffusion method. Zone diameters were measured and interpreted as susceptible, intermediate, or resistant according to CLSI M100 breakpoints criteria used by the hospital microbiology laboratory during the study period, May 28, 2023, to September 8, 2023 which is applicable to the bacterial species and antibiotic tested [12, 29]. No retrospective reinterpretation of susceptibility categories was performed using later CLSI breakpoint revisions. The preparation of the suspension of confirmed pure isolates involved selecting 3 to 5 colonies, emulsifying them in 3 to 4 mL of normal saline, and adjusting the suspension to a 0.5% McFarland standard [11]. Utilizing a sterile cotton swab, the standardized inoculum was evenly distributed on cation-adjusted Muller-Hinton agar plates (Oxoid Ltd., Basingstoke, UK), supplemented with 5% sheep blood where required for fastidious organisms, employing the lawn culture technique. After a period of 3 min, a series of standard antimicrobial disks was aseptically positioned on the inoculated plates and allowed to remain at room temperature for 15 min. All the inoculated media were incubated aerobically at 37 °C for 24 h. The zones of inhibition were measured using a ruler, and the results were subsequently interpreted as susceptible, intermediate, or resistant.

Antibiotics were analyzed according to organism-specific testing panels. For Enterobacterales, including *Escherichia coli*, *Klebsiella spp*., *Proteus mirabilis*, and *Proteus vulgaris*, the panel included nitrofurantoin (300 µg), piperacillin–tazobactam (100/10 µg), imipenem (10 µg), meropenem (10 µg), amikacin (20 µg), gentamicin (20 µg), ceftriaxone (30 µg), and a representative fluoroquinolone (ciprofloxacin; 5 µg, levofloxacin; 5 µg, ofloxacin; 5 µg) where applicable. For non-fermenting Gram-negative bacilli such as *Pseudomonas aeruginosa*, only antipseudomonal agents were interpreted, including piperacillin–tazobactam (100/10 µg), ceftazidime(30 µg) where available, carbapenems(imipenem; 10 µg, meropenem; 10 µg), aminoglycosides (amikacin; 20 µg, gentamicin; 10 µg), and a relevant fluoroquinolone (ciprofloxacin; 5 µg, levofloxacin; 5 µg, ofloxacin; 5 µg). For Gram-positive isolates, including *Staphylococcus aureus*, *Staphylococcus saprophyticus*, and *Enterococcus faecalis*, organism-appropriate urinary and systemic agents were interpreted according to applicable breakpoint tables. Antibiotics not appropriate for a given organism or specimen type were recorded as not applicable and excluded from percentage calculations. All antibiotics were procured from Abtek Biologicals, Ltd., Liverpool, UK (Supplementary File 4) [12].

#### Quality control

To ensure the accuracy of laboratory test results, a standard bacteriological procedure was employed. Quality control for culture identification and antimicrobial susceptibility testing was performed using American Type Culture Collection reference strains. (ATCC) reference strains. *Escherichia coli* ATCC 25,922, *Pseudomonas aeruginosa* ATCC 27,853, *Staphylococcus aureus* ATCC 25,212, *E. faecalis* ATCC 29,212 were used for the biochemical test validation. *Proteus mirabilis* ATCC 29,906, *Staphylococcus saprophyticus* ATCC 15,305, *Klebsiella pneumoniae* ATCC 13,883, *Proteus vulgaris* ATCC 8427 wereused to verify biochemical identification procedures, culture media performance, antimicrobial disk potency, and zone-diameter interpretation [12]. 

#### Interpretation of results

Culture results and antimicrobial susceptibility results were interpreted separately. Semi-quantitative culture criteria were used to determine clinically significant uropathogens, whereas AST interpretation was based on CLSI M100 organism-specific breakpoint tables. Only clinically significant uropathogens were included in AST analysis; mixed growth, probable contaminants, and asymptomatic bacteriuria without clinical indication were excluded [13]. AST results were expressed in the sensitive, intermediate sensitive, resistant to specific antibiotics. Only clinically significant uropathogen was considet for AST testing, while contamination or asymptomatic bacteriuria were not included. Antimicrobial susceptibility testing was performed to guide appropriate treatment decisions [12]. 

### Ethical approval

Ethical approval was obtained prior to the commencement of the study from the Institutional Review Committee of MBAHS (IRC-MBAHS) Approval number; IRC-024-079.

### Data analysis

Data were entered into Microsoft Excel, cleaned, and analysed using SPSS version 16. Descriptive statistical analysis was performed. Categorical variables were summarized as frequencies and percentages, while relevant denominators were reported separately for culture-positive isolates, isolates with complete AST records, and isolates eligible for MDR classification. No inferential statistical testing was planned because the study objective was descriptive and surveillance-oriented rather than hypothesis-testing.

## Results

Among 3,893 urine specimens processed during the study period, 1557 (40.0%) showed no bacterial growth, 1174 (30.16%) showed mixed bacterial growth or probable contamination, and 1,162 (29.84%) yielded clinically significant uropathogens. Complete AST data were available for 996 of 1,162 clinically significant isolates. Therefore, 166 isolates had incomplete or unavailable AST records and were excluded from drug-specific AST analysis. MDR classification was possible only for isolates with sufficient class-level susceptibility data; therefore, denominators vary between the overall isolate distribution, AST analysis, and MDR analysis. Among the clinically significant isolates, 788 (67.81%) were Gram-negative bacteria and 374 (32.19%) were Gram-positive bacteria. Among culture-positive patients, 907 (78.06%) were female and 255 (21.94%) were male. The highest number of culture-positive patients belonged to the 20–39 year age group (355, 30.55%), followed by the 40–59 year age group (234, 20.14%). The patients involved in this study were from different ethnicity, as such, among all the ethnic groups, majority belonged to upper caste 618 (53.18%) followed by disadvantaged Janajati 313 (26.94%). The distribution of culture-positive patients by demographic characteristics is shown in Table 1.

**Table 1:** Demographic characteristics of culture-positive patients

Characteristics	Frequency (*N* = 1162)	Percentage (%)
**Gender**		
Male	255	21.94
Female	907	78.06
**Age**		
upto 28 Days (Nb)	15	1.29
1 to 11 Month (Infancy)	35	3.01
1 to 3 Yrs. (Yc)	116	9.98
4 to 12 Yrs. (Children)	160	13.77
13 to 19 Yrs. (Adolescent)	80	6.88
20–39 Yrs. (Adult)	355	30.55
40–59 Yrs. (Old Adult)	234	20.14
60 to 70 Yrs.	100	8.61
More than 71	67	5.77
**Ethnic Group**		
Dalit	82	7.06
Disadvantage Janajati	313	26.94
Disadvantage Non-Dalit	71	6.11
Religious Minority	9	0.78
Relative advanced Janajati	69	5.93
Upper Caste	618	53.18

Nb: New born, Yc: Young child; Note: The categorization of age and ethnic group are done on the basis of Nepal Health Research Council, Nepal

### Isolated bacterial frequency distribution

Out of 3893 patients’ sample, 1162 (29.84%) bacterial isolates were detected among which gram-negative bacteria accounted for majority of the isolates 788 (67.81%) while gram positive accounted for one third of isolates 374 (32.19%). Out of 1162 isolates the most common isolate was of *E. coli* 548 (47.16%) followed by *Klebsiella sps.* 169 (14.54%). Among the gram-positive bacteria *Enterococcus faecalis* 138 (11.88%) was the most prevalent (Table 2). We have presented the demographic characteristics and the isolated uropathogens distribution patterns as Supplementary File 1.

**Table 2:** Isolated bacterial frequency distribution

Characteristics	Frequency(*N* = 1162)	Percentage (%)
**Type of Bacterial Isolates**		
Gram Negative Bacteria	788	67.81
Gram Positive Bacteria	374	32.19
**Bacterial Isolate**		
***Gram negative bacteria***		
*Escherichia coli* ^*^	548	47.16
*Proteus mirabilis* ^*^	21	1.81
*Klebsiella sps.* ^*^	169	14.54
*Pseudomonas aeruginosa* ^*^	27	2.32
*Proteus vulgaris* ^*^	15	1.29
***Gram positive bacteria***		
*Enterococcus faecalis* ^*#*^	138	11.88
*Staphylococcus saprophyticus* ^*#*^	106	9.12
*Staphylococcus aureus* ^*##*^	138	11.88

Note: Gram-positive bacteria^#^, Gram negative bacteria^*^

### AST of commonly tested antibiotics on various pathogens

A total of 996 pathogenic bacterial isolates with complete antimicrobial susceptibility data were included in the AST analysis (Supplementary File 2). Drug-specific resistance rates were interpreted cautiously in relation to published empirical UTI treatment thresholds and local stewardship principles. Antibiotics with high local resistance rates were not recommended for empirical use unless culture and susceptibility results supported their use. These interpretations were made only for organism–antibiotic combinations that were clinically relevant and supported by applicable AST breakpoint guidance. A resistance rate of 1–10% is considered low and supports empirical therapy. Rates between 11 and 20% indicate moderate resistance, where empirical use should be considered with caution. When resistance is 21% or higher, empirical use is discouraged due to the high risk of treatment failure.

When analyzed by drug class Nitrofuran group, nitrofurantoin was highly susceptible to *P. vulgaris* isolates (92.90%), followed by *S. saprophyticus* (81.40%) and *P. mirabilis* (76.20%). However, *E. faecalis* (5.00%) and *Staphylococcus aureus* (7.60%) exhibited considerable resistance to Nitrofurantoin. Among the Penicillins, *P. aeruginosa* showed the highest sensitivity (55.00%) and *E. faecalis* depicted a moderate response (31.70%) to the Piperacillin. Carbapenems displayed superior antibacterial activity, especially meropenem, *P. mirabilis* (66.70%) showed high sensitive to Meropenem followed by *P. vulgaris* (57.10%).

Within the Aminoglycoside class, all organism showed a sensitivity above 50% where *P. vulgaris* showed the highest sensitivity (92.90%) followed by *Klebsiella sps.* (87.20%) to the Amikacin. Among the Cephalosporins, showed high resistance rate (92.90%) for *P. vulgaris*. Ceftriaxone was found to be sensitive majorly for *S. saprophyticus* (52.90%) followed by *Klebsiella sps.* (51.00%). *P. mirabilis* showed the highest resistance (33.3.%) for ceftriaxone followed by *P. vulgaris* (28.60%).

The Fluoroquinolones showed varied susceptibility level, with *E. coli* demonstrating highly susceptibility to Levofloxacin (54.40%). Likewise, the highest resistance to Levofloxacin (40.00%) followed by *P. vulgaris* (35.70%). Linezolid and chloramphenicol were majorly not tested for several organisms (Supplementary File 2).

### Antibiotics resistance profile of bacterial isolates

Antibiotic resistance profiles were categorized based on the number of antimicrobial classes to which isolates were resistant. Resistance to one (One DR) and two (Two DR) antimicrobial classes was reported descriptively, while multidrug resistance (MDR) was defined as resistance to three or more antimicrobial classes. While One DR and Two DR categories are presented for descriptive purposes, MDR represents the clinically meaningful resistance outcome. These categories are not independent and were not combined for inferential analysis. *E. coli* isolates revealed that 18.40% were categorized as MDR. *P. mirabilis* showed a comparatively greater percentage of MDR isolates (18.75%). Among the isolates of *Enterococcus faecalis*, 17.19% were MDR. The MDR incidence for *Staphylococcus saprophyticus* was serious, with 24.44% of the cases of MDR. Likewise, 13.46% of *Klebsiella sps.* isolates were found to be MDR. Among the Gram-positive *Staphylococcus aureus*, 26.88% of isolates were MDR. In *Pseudomonas aeruginosa*, 38.90% of isolates were MDR. *Proteus vulgaris* had the highest MDR rate among all the tested species, with 46.15% of the isolates falling into this category (Table 3**).** We have presented the antibiotic resistance pattern with demographic variables as Supplementary File 3.

**Table 3:** Antibiotics resistance profile of bacterial isolates

Bacterial Isolates	One DR		Two DR		MDRs		Total (*n*, %)
	*N*	%	*N*	%	*N*	%	
*E. coli*	160	46.80%	119	34.80%	63	18.40%	342 (46.40)
*P. mirabilis*	5	31.25%	8	50.00%	3	18.75%	16 (2.17)
*E. faecalis*	34	53.12%	19	29.69%	11	17.19%	64 (8.55)
*S. saprophyticus*	37	41.11%	31	34.45%	22	24.44%	90 (12.21)
*Klebsiella sps.*	53	50.97%	37	35.58%	14	13.46%	104 (14.11)
*Staphylococcus aureus*	30	32.26%	38	40.86%	25	26.88%	93 (12.35)
*P. aeruginosa*	8	44.45%	3	16.67%	7	38.90%	18 (2.44)
*P. vulgaris*	4	30.77%	3	23.08%	6	46.15%	13 (1.76)
Total	331 (44.73)		258 (34.86)		151 (20.40)		*N* = 740

(Note: One DR- “Resistance to one antimicrobial class”, Two DR- “Resistance to two antimicrobial classes”, MDR- “Resistance to three or more antimicrobial classes”)

## Discussion

This study describes the distribution of urinary bacterial isolates and antimicrobial susceptibility patterns at a single provincial hospital in Nepal. The findings are relevant to local antimicrobial stewardship and are contextually aligned with national AMR policy discussions; however, they should not be interpreted as direct evidence of progress toward National Action Plan implementation or as representative of provincial or national AMR surveillance. However, antimicrobial susceptibility patterns are dynamic, this finding is interpreted as a facility-level baseline for the study period rather than as a current resistance estimate. Periodic updating of the hospital urine antibiogram is necessary before using these data for ongoing empirical treatment decisions.

Hetauda Hospital is a provincial-based hospital located in Hetauda, Bagmati Province, Nepal, [13] and encounters a daily average of 800 patients. The hospital has its own dedicated laboratory section that conducts biochemical, haematological, and pathological tests through a team of laboratory officers, technicians, and microbiologists. The hospital is located within the national highway, making it geographically nearer and accessible to patients from the western and southern parts of the country [14] thus, this hospital represents with high volume and diverse patient population.

A total of 29.84% of urine specimens yielded bacterial isolates. Gram-negative organisms predominated (67.81%), while gram-positive organisms accounted for 32.19% of isolates. *Escherichia coli* was the most common isolate. Although the predominance of gram-negative uropathogens is consistent with previous reports, the proportion of gram-positive isolates should be interpreted cautiously because clean-catch urine specimens may be affected by contamination and the dataset did not include contamination indicators or repeat-culture confirmation. Cases of bacterial isolates were also observed in the literature from Nepal, predominantly from Kathmandu Valley [15–19], with gram-negative bacteria being the major ones.

In our study, descriptive differences in resistance across age groups were observed. However, these findings should be interpreted cautiously because the analysis did not adjust for prior hospitalization, catheter use, previous antibiotic exposure, comorbidity, or distinction between community- and hospital-acquired infection. Accordingly, the age-related observations are exploratory and hypothesis-generating [20]. In contrast, pediatric isolates showed relatively lower resistance trends, though emerging data point to significant age-dependent susceptibility differences among very young children [21]. These findings highlight the importance of incorporating demographic characteristics such as age into antimicrobial resistance surveillance, as such factors can significantly influence resistance dynamics and guide more targeted interventions. In the present study, *Klebsiella* isolates were reported collectively at the genus level because species-level differentiation was not consistently available in the laboratory records, and *Staphylococcus aureus* was distinctly presented apart from *Coagulase-negative staphylococci*.

Our study showed that 342 (46.40%) *E. coli* cases were resistant to drugs, followed by *Klebsiella sps.* (14.11%). Another study revealed that 64.90% of *E. coli* isolates were multidrug-resistant (MDR), and 38.9% produced *Extended spectrum beta-lactamase* (ESBL) [22]. A total prevalence of 20.35% (150 cases) were found to be MDR, thus depicting an alarming scenario. Our result of MDR cases was in contrast with cases from the territory hospital due to varying factors of sample size and only focusing on a specific group of bacteria [16–18]. 

NAP has also reported that the commonly isolated bacteria in Nepalese context are getting resistant to various group of antibiotics belonging to aminoglycosides, penicillins, fluoroquinolones and polymixins [6]. Our study also found a wide range of resistance patterns among cephalosporins and fluoroquinolones. The observed resistance to commonly used cephalosporins and fluoroquinolones suggests that these agents should be used cautiously for empirical treatment of suspected UTI in this hospital setting unless supported by culture and susceptibility results. The relatively higher susceptibility observed for selected agents, including amikacin in several Gram-negative isolates, indicates potential therapeutic options for resistant infections; however, such agents should be selected according to infection severity, site of infection, patient factors, drug safety, and antimicrobial stewardship principles. Therefore, the present findings are best interpreted as supporting AST-guided prescribing and periodic local antibiogram development rather than identifying a universally preferred antibiotic. Since the major reasons could be the rampant use of such antibiotics from the pharmacies and use by patients without the check-up with doctors, further leading to AMR cases [23]. Literatures also shows the increasing use of such drugs as growth feeders and also for therapeutic use in poultry, further underscoring the need to educate farmers to promote the rational use of antibiotics [24]. Awaring public on prescription antibiotics, and continuous professional development programs regarding antibiotic awareness is necessary to mitigate such increasing use of drugs [25]. Along with that, hospitals could also focus on its development of hospital formulary focusing on antibiotics with evidence, AWaRe group of antibiotics classification, inclusion of national surveillance data so as to help prescriber make effective medication management decisions. NAP highlights the fact that it is essential to strengthen the surveillance system to cover a wide scope of samples from human, animal and even food sources to further align the goals with OHA [6]. One example to limit the use of fluoroquinolone can also been seen through the National Antimicrobial Treatment Guideline 2023 where the guideline advises the prescribers and healthcare professionals on limiting the use of fluoroquinolone because of the increasing prevalence of MDR Tuberculosis [26]. However, the question for its acceptance, practicality and understanding by several healthcare professionals needs to be checked.

Literatures also focuses on the fact that resistance over the period further increases the use of higher form of antibiotics including watch and reserve group (From the AWaRe) further leading to increased cost and expenses [27, 28]. This increasing cases of resistance of antibiotic in case of Nepal could be due to the use of antibiotics as self-medication and due to the easily availability of such drugs over the coutners [25, 29]. 

Similarly, Nepal’s own National Antimicrobial Treatment Guidelines, 2023, also aim to regulate and address the emerging issue of AMR along with the rational use of antimicrobial agents by providing evidence-based information to prescribers on selecting proper antimicrobials [26]. The guideline also incorporating the antibiogram from national AMR surveillance data along with several considerations such as inclusion of availability and affordability of antimicrobial agents in reference to national (National List of Essential Medicine) and international guidelines (WHO AWaRe classification) [26].

### Strengths and limitations

The study findings can act as a urine antibiogram for hospital inpatient and outpatient settings to determine the rational use of antibiotics in association with the National Antimicrobial Treatment Guideline 2023, which will serve as a quality improvement project for hospitals. Another major strength is the interconnection with the Ministry of Health and Health Directorate in strengthening the surveillance facility within the Bagmati Province. The data can also be used within the Hetauda Sub-metropolitan city’s primary and private hospitals involved in treating infectious diseases. The relatively large number of isolates analysed is a major strength, providing robust resistance pattern data. For AST analysis, complete records were available for 996 of 1,162 clinically significant isolates, while 166 isolates had incomplete AST data due to retrospective record limitations and non-uniform routine testing panels were also limitation for this study. Likewise, *Klebsiella* species were reported collectively due to the equipment limitation. Despite the generalizability in terms of bacterial isolates, this study does not explain the scenario of other microbial isolates, such as viruses, fungi, parasites, and their susceptibility patterns. Furthermore, it was single-center, included urine specimens only, used a cross-sectional retrospective design, lacked complete AST data for some isolates. *Klebsiella* isolates were reported collectively at the genus level because species-level documentation was incomplete. In addition, confirmatory testing for *methicillin-resistant Staphylococcus aureus* (MRSA), extended-spectrum beta-lactamase (ESBL) production, and beta-lactamase production was not routinely available in the retrospective dataset. Therefore, the MDR findings should not be interpreted as MRSA or ESBL prevalence estimates. Accordingly, the findings should be interpreted as local facility-level data and should not be generalized to provincial or national AMR surveillance. Prior antibiotic exposure before urine specimen collection was not consistently recorded; therefore, its effect on culture positivity and resistance patterns could not be assessed. Multicenter studies using standardized methods and broader specimen inclusion are needed for wider inference. As with other retrospective laboratory-based surveillance studies, independent reproducibility of the original specimen collection, culture processing, and AST results cannot be fully confirmed. However, the hospital laboratory followed standard bacteriological procedures, and quality-control strains were used to support the reliability of routine culture and susceptibility testing. Sex-specific denominator data for all urine specimens submitted during the study period were not consistently available; therefore, sex-specific positivity rates could not be calculated. Likewise, treatment prescriptions and follow-up outcomes were not available in the laboratory records, this study cannot determine whether AST-guided therapy resulted in clinical cure or microbiological clearance.

## Conclusion

This single-center retrospective study provides recent facility-level urine culture and AST data for the period May 28, 2023, to September 8, 2023 from a provincial hospital in Nepal. Nearly one-third of urine specimens yielded clinically significant bacterial uropathogens, with Gram-negative bacteria predominating and *Escherichia coli* being the leading isolate. Considerable resistance to selected cephalosporins and fluoroquinolones supports the need for culture-guided prescribing, periodic urine antibiogram updates, and local antimicrobial stewardship. Because the study was urine-only, retrospective, and conducted at one hospital, the findings should be interpreted as local facility-level evidence rather than provincial or national AMR surveillance estimates. The findings provide a baseline for local antimicrobial stewardship but require periodic updating because antibiotic susceptibility patterns may change over time.

## Supplementary Information

Below is the link to the electronic supplementary material.

Supplementary Material 1: Demographic characteristic of participants.

Supplementary Material 2: Antimicrobial Susceptibility test.

Supplementary Material 3: Antibiotic Resistance Pattern.

Supplementary Material 4: Some representative disc diffusion image.

## Data Availability

All data relevant to the study are included in the article or uploaded as supplementary information.
