# Supplementary material for: Urine antibiogram and antimicrobial susceptibility pattern in a provincial hospital of Nepal: a cross-sectional study
Source: BMC Infect Dis. 2026 Jun 8;26:1488. doi: 10.1186/s12879-026-13781-x (PMC13474701; doi:10.1186/s12879-026-13781-x)
Supplement: Supplementary file 1 [file 12879_2026_13781_MOESM1_ESM.docx]

**Supplementary file-1: Demographic characteristics and the isolated organism’s distribution patterns**

|  | Isolate_Uropathogen_Name | | | | | | | | Total |
| --- | --- | --- | --- | --- | --- | --- | --- | --- | --- |
|  | *E.coli* | *P.mirabilis* | *E. faecalis* | *S. saprophyticus* | *Klebsiella sps* | *Staph.species* | *P. aeruginosa* | *P. vulgaris* |  |
| **Gender** |  |  |  |  |  |  |  |  |  |
| Male | 122 | 2 | 40 | 15 | 37 | 32 | 5 | 2 | 255 |
| Female | 426 | 19 | 98 | 91 | 132 | 106 | 22 | 13 | 907 |
| **Age** |  |  |  |  |  |  |  |  |  |
| upto 28 Days(Nb) | 9 | 0 | 0 | 1 | 3 | 1 | 1 | 0 | 15 |
| 1 to 11 Month(Infancy) | 22 | 0 | 6 | 2 | 2 | 2 | 0 | 1 | 35 |
| 1 to 3 Yrs(Yc) | 52 | 0 | 24 | 8 | 15 | 13 | 3 | 1 | 116 |
| 4 to 12 Yrs(Childern) | 80 | 1 | 25 | 8 | 20 | 21 | 4 | 1 | 160 |
| 13 to 19 Yrs(Adolescent) | 35 | 0 | 11 | 2 | 17 | 11 | 4 | 0 | 80 |
| 20-39 Yrs(Adult) | 164 | 5 | 41 | 54 | 52 | 32 | 4 | 3 | 355 |
| 40-59 Yrs(Old Adult) | 104 | 10 | 17 | 20 | 35 | 36 | 4 | 8 | 234 |
| 60 to 70 Yrs | 49 | 2 | 9 | 4 | 12 | 17 | 6 | 1 | 100 |
| More than 71 | 33 | 3 | 5 | 7 | 13 | 5 | 1 | 0 | 67 |
