# Supplementary material for: Urine antibiogram and antimicrobial susceptibility pattern in a provincial hospital of Nepal: a cross-sectional study
Source: BMC Infect Dis. 2026 Jun 8;26:1488. doi: 10.1186/s12879-026-13781-x (PMC13474701; doi:10.1186/s12879-026-13781-x)
Supplement: Supplementary file 2 [file 12879_2026_13781_MOESM2_ESM.docx]

**Supplementary File 2. Antimicrobial Susceptibility test of commonly tested antibiotics on various pathogens**

|  | | **Isolate Uropathogen, 996 (%)** | | | | | | | |
| --- | --- | --- | --- | --- | --- | --- | --- | --- | --- |
|  |  | ***Gram negative bacteria*** | | | | | ***Gram positive bacteria*** | | |
| **Antibiotics** | **AST** | ***E. coli*** | ***P. mirabilis*** | ***P. aeruginosa*** | ***P. vulgaris*** | ***Klebsiella sps.*** | ***Staphylococcus* *aureus*** | ***E. faecalis*** | ***S. saprophyticus*** |
| **Nitrofuran** | | | | | | | | | |
| Nitrofurantoin | NT | 12.90 | 19.00 | 15.00 | 7.10 | 11.40 | 19.30 | 19.00 | 5.90 |
|  | S | 69.20 | 76.20 | 40.00 | 92.90 | 67.80 | 59.70 | 54.00 | 81.40 |
|  | IM | 13.80 | 4.80 | 45.00 | - | 16.10 | 13.40 | 22.00 | 9.80 |
|  | R | 4.10 | - | - | - | 4.70 | 7.60 | 5.00 | 2.90 |
| **Penicillin** | | | | | | | | | |
| Piperacillin and Tazobactam | NT | 78.90 | 100.00 | 45.00 | 100.00 | 76.50 | 79.70 | 68.30 | 93.10 |
|  | S | 21.10 | - | 55.00 | - | 23.50 | 19.50 | 31.70 | 6.90 |
|  | IM | - | - | - | - | - | 0.80 | - | - |
| **Carbapenem** | | | | | | | | | |
| Imipenem | NT | 99.10 | 100.00 | 95.00 | 100.00 | 98.00 | 100.00 | 94.10 | 100.00 |
|  | S | 0.90 | - | 5.00 | - | 2.00 | - | 5.90 | - |
| Meropenem | NT | 67.00 | 33.30 | 95.00 | 42.90 | 79.90 | 98.30 | 96.00 | 87.30 |
|  | S | 33.00 | 66.70 | 5.00 | 57.10 | 20.10 | 0.80 | 4.00 | 12.70 |
|  | R | - | - | - | - | - | 0.80 | - | - |
| **Aminoglycosides** | | | | | | | | | |
| Amikacin | NT | 13.30 | 19.00 | 35.00 | 7.10 | 9.50 | 88.20 | 15.50 | 10.70 |
|  | S | 83.90 | 76.20 | 65.00 | 92.90 | 87.20 | 79.30 | 52.50 | 86.40 |
|  | IM | 2.40 | 4.80 | - | - | 3.40 | 2.50 | 1.90 | 2.90 |
|  | R | 0.40 | - | - | - | - | - | - | - |
| Gentamycin | NT | 93.30 | 100.00 | 80.00 | 92.90 | 95.30 | 95.80 | 95.00 | 98.00 |
|  | S | 6.70 | - | 20.00 | 7.10 | 4.70 | 3.40 | 2.00 | 2.00 |
|  | IM | - | - | - | - | - | 0.80 | - | - |
|  | R | - | - | - | - | - | - | 3.00 | - |
| **Cephalosporins** | | | | | | | | | |
| Cefixime | NT | 13.20 | 19.00 | 10.00 | 7.10 | 18.80 | 22.70 | 27.70 | 8.80 |
|  | S | 3.50 | - | 5.00 | - | 2.00 | 2.50 | 2.00 | 1.00 |
|  | IM | 12.30 | 9.50 | - | - | 15.40 | 4.20 | 8.90 | 3.90 |
|  | R | 71.10 | 71.40 | 85.00 | 92.90 | 63.80 | 70.60 | 61.40 | 86.30 |
| Ceftriaxone | NT | 12.90 | 19.00 | 20.00 | 7.10 | 14.10 | 14.40 | 20.80 | 9.80 |
|  | S | 49.10 | 42.90 | 35.00 | 50.00 | 51.00 | 48.30 | 47.50 | 52.90 |
|  | IM | 24.40 | 4.80 | 40.00 | 14.30 | 22.10 | 22.90 | 20.80 | 23.50 |
|  | R | 13.60 | 33.30 | 5.00 | 28.60 | 12.80 | 14.40 | 10.90 | 13.70 |
| **Fluoroquinolones** | | | | | | | | | |
| Ciprofloxacin | NT | 97.00 | 100.00 | 85.00 | 100.00 | 94.70 | 93.20 | 95.00 | 99.00 |
|  | S | 1.50 | - | 10.00 | - | 4.00 | 5.10 | 4.00 | - |
|  | IM | 0.20 | - | 5.00 | - | 0.70 | 1.70 | - | - |
|  | R | 1.30 | - | - | - | 0.70 | - | 1.00 | 1.00 |
| Levofloxacin | NT | 10.40 | 19.00 | 5.00 | 21.40 | 12.10 | 14.30 | 18.80 | 6.90 |
|  | S | 54.40 | 47.60 | 40.00 | 28.60 | 55.70 | 36.10 | 54.50 | 38.20 |
|  | IM | 22.50 | 14.30 | 15.00 | 14.30 | 24.20 | 30.30 | 18.80 | 36.30 |
|  | R | 12.70 | 19.00 | 40.00 | 35.70 | 8.10 | 19.30 | 7.90 | 18.60 |
| Ofloxacin | NT | 11.40 | 19.00 | - | 7.10 | 12.10 | 15.10 | 17.60 | 8.80 |
|  | S | 39.20 | 28.60 | 40.00 | 7.10 | 42.30 | 23.50 | 41.20 | 21.60 |
|  | IM | 20.50 | 23.80 | 15.00 | 35.70 | 20.80 | 18.50 | 23.50 | 27.50 |
|  | R | 28.90 | 28.60 | 45.00 | 50.00 | 24.80 | 42.90 | 17.60 | 42.20 |
| **Oxazolidinones** | | | | | | | | | |
| Linezolid | NT | 100.00 | 100.00 | 100.00 | 100.00 | 100.00 | 100.00 | 100.00 | 97.10 |
|  | S | - | - | - | - | - | - | - | 2.90 |
| **Chloramphenicol** | | | | | | | | | |
| Chloramphenicol | NT | 99.20 | 100.00 | 100.00 | 100.00 | 98.00 | 98.30 | 97.00 | 99.00 |
|  | S | 0.60 | - | - | - | 1.30 | 1.70 | 2.00 | - |
|  | IM | 0.20 | - | - | - | - | - | - | - |
|  | R | - | - | - | - | 0.70 | - | 1.00 | 1.00 |

Note: This table highlights the antibiotic susceptibility testing of several antibiotics over microorganism. Percentages indicate the proportion of isolates classified and here NT means “Not tested”, S means “Sensitive”, IM means “Intermediate Resistant”, R stands for “Resistant” and - indicates “None
