# Supplementary material for: Urine antibiogram and antimicrobial susceptibility pattern in a provincial hospital of Nepal: a cross-sectional study
Source: BMC Infect Dis. 2026 Jun 8;26:1488. doi: 10.1186/s12879-026-13781-x (PMC13474701; doi:10.1186/s12879-026-13781-x)
Supplement: Supplementary file 3 [file 12879_2026_13781_MOESM3_ESM.docx]

**Supplementary file-3: Antibiotic Resistance Pattern with Demographic Variables**

| **Variables** | ***Antibiotics Resistance Profile of Bacterial Isolates*** | | | **Total** |
| --- | --- | --- | --- | --- |
|  | **One DR** | **Two DR** | **MDR** |  |
| Gender | | | | |
| Male | 80 | 51 | 27 | 158 |
| Female | 251 | 207 | 124 | 582 |
| *Total* | 331 | 258 | 151 | 740 |
| **Age** | | | | |
| upto 28 Days (Nb) | 5 | 3 | 0 | 8 |
| 1 to 11 Month (Infancy) | 11 | 9 | 2 | 22 |
| 1 to 3 Yrs (Young child) | 39 | 17 | 4 | 60 |
| 4 to 12 Yrs (Children) | 67 | 27 | 6 | 100 |
| 13 to 19 Yrs (Adolescent) | 31 | 17 | 4 | 52 |
| 20-39 Yrs (Adult) | 99 | 92 | 36 | 227 |
| 40-59 Yrs (Old Adult) | 48 | 55 | 51 | 154 |
| 60 to 70 Yrs | 17 | 24 | 24 | 65 |
| More than 71 | 14 | 14 | 24 | 52 |
| *Total* | 331 | 258 | 151 | 740 |
| **Ethnic Group** | | | | |
| Dalit | 19 | 20 | 10 | 49 |
| Disadvantage Janajati | 87 | 74 | 37 | 198 |
| Disadvantage Non-Dalit | 20 | 21 | 8 | 49 |
| Religious Minority | 5 | 1 | 1 | 7 |
| Relative advanced Janajati | 23 | 14 | 8 | 45 |
| Upper Caste | 177 | 128 | 87 | 392 |
| *Total* | 331 | 258 | 151 | 740 |

*(Note: One DR- “Resistance to one antimicrobial class”, Two DR- “Resistance to two antimicrobial classes”, MDR- “Resistance to three or more antimicrobial classes”)*
