# Supplementary material for: Urine antibiogram and antimicrobial susceptibility pattern in a provincial hospital of Nepal: a cross-sectional study
Source: BMC Infect Dis. 2026 Jun 8;26:1488. doi: 10.1186/s12879-026-13781-x (PMC13474701; doi:10.1186/s12879-026-13781-x)
Supplement: Supplementary file 4 [file 12879_2026_13781_MOESM4_ESM.docx]

**Supplementary File-4: Image of the some of the disc diffusion assay**

*Pseudomonas aureginosa with tested antibiotics*

*Pseudomonas aureginosa with tested antibiotics*

*E. coli with tested antibiotics*

*Klebsiella sps. with tested antibiotics*

*E. coli with tested antibiotics*

*S. aureus and E. coli with tested antibiotics*

*S. aureus and E. coli with tested antibiotics*
